# 3-{2-[2-(3-Hy­droxy­benzyl­idene)hydrazin-1-yl]-1,3-thia­zol-4-yl}-2*H*-chromen-2-one hemihydrate

**DOI:** 10.1107/S1600536810019653

**Published:** 2010-05-29

**Authors:** Afsheen Arshad, Hasnah Osman, Kit Lam Chan, Jia Hao Goh, Hoong-Kun Fun

**Affiliations:** a School of Chemical Sciences, Universiti Sains Malaysia, 11800 USM, Penang, Malaysia; b School of Pharmaceutical Sciences, Universiti Sains Malaysia, 11800 USM, Penang, Malaysia; c X-ray Crystallography Unit, School of Physics, Universiti Sains Malaysia, 11800 USM, Penang, Malaysia

## Abstract

In the title compound, C_19_H_13_N_3_O_3_S·0.5H_2_O, both organic mol­ecules (*A* and *B*) exist in *E* configurations with respect to the acyclic C=N bond and have similar overall conformations. In mol­ecule *A*, the essentially planar thia­zole ring [maximum deviation = 0.010 (2) Å] is inclined at inter­planar angles of 11.44 (10) and 32.50 (12)°, with the 2*H*-chromene ring system and the benzene ring, respectively. The equivalent values for mol­ecule *B* are 0.002 (2) Å, 7.71 (9) and 12.51 (12)°. In the crystal structure, neighbouring mol­ecules are inter­connected into infinite layers lying parallel to (010) by O—H⋯O, O—H⋯N, N—H⋯O and C—H⋯O hydrogen bonds. Further stabilization of the crystal structure is provided by weak inter­molecular C—H⋯π and π–π [centroid–centroid distance = 3.6380 (19) Å] inter­actions.

## Related literature

For general background to and applications of amino­thia­zoles, see: Anderson *et al.* (2002 ▶); Finn *et al.* (2004 ▶); Gursoy & Karah (2000 ▶); Habib & Khalil (1984 ▶); Hiremath *et al.* (1992 ▶); Hofmanová *et al.* (1998 ▶); Jayashree *et al.* (2005 ▶); Karah *et al.* (1998 ▶); Kimura *et al.* (1985 ▶); Laffitte *et al.* (2002 ▶); Mitscher (2002 ▶); Moffett (1964 ▶); Ohkuba *et al.* (1995 ▶); Patt *et al.* (1992 ▶); Tassies *et al.* (2002 ▶); Wattenberg *et al.* (1979 ▶); Weber *et al.* (1998 ▶). For the preparation of the title compound, see: Lv *et al.* (2010 ▶); Siddiqui *et al.* (2009 ▶). For related structures, see: Arshad *et al.* (2010**a* ▶,b* ▶). For the stability of the temperature controller used for the data collection, see: Cosier & Glazer (1986 ▶).
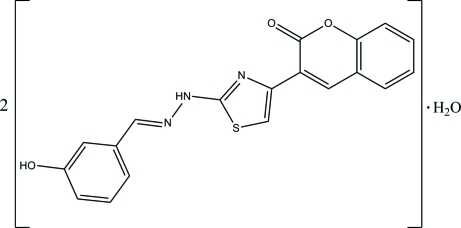


## Experimental

### 

#### Crystal data

C_19_H_13_N_3_O_3_S·0.5H_2_O
*M* *_r_* = 372.39
Monoclinic, 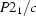

*a* = 8.012 (3) Å
*b* = 32.775 (11) Å
*c* = 12.619 (4) Å
β = 93.034 (7)°
*V* = 3309 (2) Å^3^
*Z* = 8
Mo *K*α radiation
μ = 0.23 mm^−1^
*T* = 100 K
0.34 × 0.14 × 0.05 mm

#### Data collection

Bruker APEXII DUO CCD diffractometer
Absorption correction: multi-scan (*SADABS*; Bruker, 2009 ▶) *T*_min_ = 0.928, *T*_max_ = 0.990
31194 measured reflections
7564 independent reflections
5266 reflections with *I* > 2σ(*I*)
*R*_int_ = 0.073

#### Refinement

*R*[*F* ^2^ > 2σ(*F* ^2^)] = 0.048
*wR*(*F* ^2^) = 0.128
*S* = 1.06
7564 reflections
480 parameters
H-atom parameters constrained
Δρ_max_ = 0.94 e Å^−3^
Δρ_min_ = −0.31 e Å^−3^

### 

Data collection: *APEX2* (Bruker, 2009 ▶); cell refinement: *SAINT* (Bruker, 2009 ▶); data reduction: *SAINT*; program(s) used to solve structure: *SHELXTL* (Sheldrick, 2008 ▶); program(s) used to refine structure: *SHELXTL*; molecular graphics: *SHELXTL*; software used to prepare material for publication: *SHELXTL* and *PLATON* (Spek, 2009 ▶).

## Supplementary Material

Crystal structure: contains datablocks global, I. DOI: 10.1107/S1600536810019653/hb5467sup1.cif

Structure factors: contains datablocks I. DOI: 10.1107/S1600536810019653/hb5467Isup2.hkl

Additional supplementary materials:  crystallographic information; 3D view; checkCIF report

## Figures and Tables

**Table 1:** Hydrogen-bond geometry (Å, °) *Cg*1 is the centroid of C14*A*–C19*A* benzene ring.

*D*—H⋯*A*	*D*—H	H⋯*A*	*D*⋯*A*	*D*—H⋯*A*
O3*A*—H3*OA*⋯O3*B*^i^	0.82	2.00	2.808 (3)	170
N2*A*—H2*NA*⋯O1*W*^ii^	0.88	1.93	2.790 (3)	167
O3*B*—H3*OB*⋯O2*B*^iii^	0.82	1.93	2.726 (3)	165
N2*B*—H2*NB*⋯O2*A*^iv^	0.84	2.05	2.878 (3)	170
O1*W*—H1*W*1⋯N1*B*^v^	0.87	2.05	2.888 (3)	161
O1*W*—H2*W*1⋯N1*A*^vi^	0.88	2.15	2.913 (3)	145
C8*A*—H8*A*⋯O1*W*^vii^	0.93	2.60	3.451 (3)	153
C5*B*—H5*B*⋯*Cg*1^iv^	0.93	2.95	3.708 (3)	139

Symmetry codes: (i) 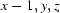; (ii) 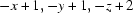; (iii) 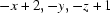; (iv) 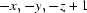; (v) 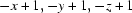; (vi) 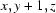; (vii) 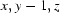.

**Fig. 1.**
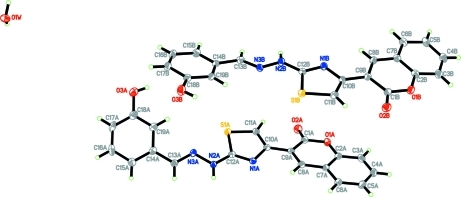
The molecular structure of (I), showing 50 % probability displacement ellipsoids for non-H atoms.

**Fig. 2.**
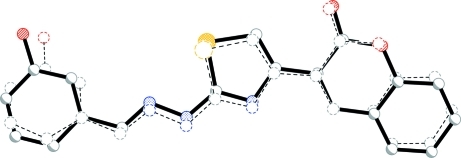
Fit of molecule A (dashed lines) on molecule B (solid lines). H atoms have been omitted for clarity.

**Fig. 3.**
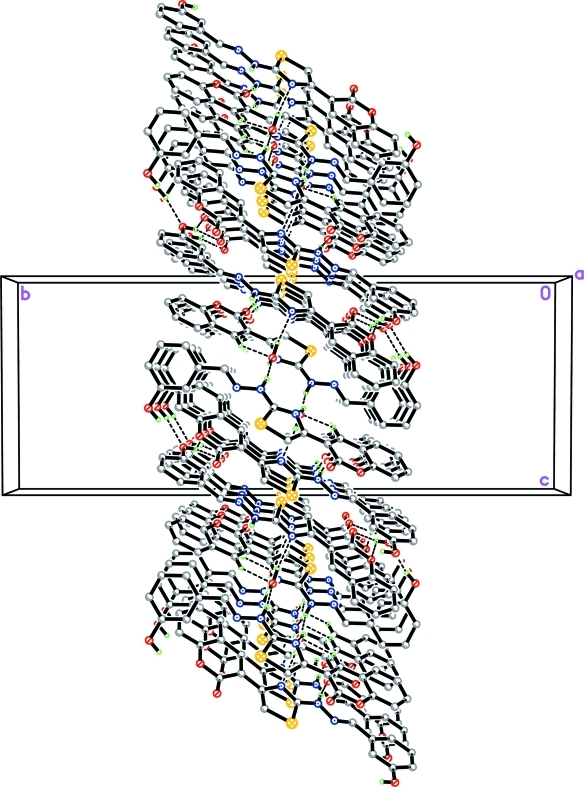
The crystal structure of (I), viewed along the a axis, showing a two-dimensional infinite network parallel to the (010) plane. H atoms not involved in intermolecular interactions (dashed lines) have been omitted for clarity.

## supplementary crystallographic information

### Comment

The biological activity of aminothiazoles is well documentated. Some of these compounds exhibit very good anti-fungal (Hiremath *et al.*, 1992), anti-bacterial (Habib & Khalil, 1984), anti-tuberculosis (Gursoy & Karah, 2000; Karah *et al.*, 1998) and anti-tumor (Wattenberg *et al.*, 1979) activities. They also have broad applications in the treatment of allergies, Schizophrenia (Ohkuba *et al.*, 1995), inflammation (Jayashree *et al.*, 2005) and hypertension (Patt *et al.*, 1992). Besides that, coumarin and its derivatives also possess significant anti-bacterial (Mitscher, 2002; Laffitte *et al.*, 2002), anti-fungal (Moffett, 1964) and cytotoxic (Weber *et al.*, 1998) activities. They also have pronounced medicinal value as anti-coagulants (Anderson *et al.*, 2002; Tassies *et al.*, 2002), free radical scavengers (Finn *et al.*, 2004), lipoxygenese and cyclooxygenese inhibitors (Kimura *et al.*, 1985, Hofmanová *et al.*, 1998). The title compound, (I) was synthesized by incorporating aminothioazole moiety to a coumarin skeleton and here we present its crystal structure.

The asymmetric unit of the title compound (Fig. 1) comprises of two crystallographically independent 3-{2-[2-(3-hydroxybenzylidene)hydrazinyl]thiazol-4-yl}-2*H*-chromen-2-one molecules and a water molecule of crystallization. Both of the independent molecules exist in *cis* configurations with respect to the acyclic N3═C13 double bond. A superposition of the non-H atoms of molecules *A* and *B* (Fig. 2) using *XP* in *SHELXTL* (Sheldrick, 2008), gave an r.m.s. deviation of 0.447 Å.

In each molecule, the thiazole ring (C10/C11/S1/C12/N1) is essentially planar, with maximum deviations of -0.010 (2) and 0.002 (2) Å, respectively, for atoms C11A of molecule *A* and C12B of molecule *B*. In molecule *A*, the thiazole ring is inclined at interplanar angles of 11.44 (10) and 32.50 (12)°, respectively, with respect to the 2*H*-chromene ring system (C1A-C9A/O1A) and C14A-C19A benzene ring; the comparable angles for molecule *B* are 7.71 (9) and 12.51 (12)°, respectively. The bond lengths and angles are comparable to those observed in closely related structures (Arshad *et al.*, 2010*a,b*).

In the crystal structure (Fig. 3), neighbouring molecules are interconnected into two-dimensional infinite networks parallel to the (010) plane by intermolecular O3A—H3OA···O3B, N2A—H2NA···O1W, O3B—H3OB···O2B, N2B—H2NB···O2A, O1W—H1W1···N1B, O1W—H2W1···N1A and C8A—H8A···O1W hydrogen bonds (Table 1). The crystal structure is further stabilized by weak intermolecular C5B—H5B···*Cg*1 interactions (Table 1) involving the centroid of C14A—C19A benzene ring as well as *Cg*2···*Cg*3 aromatic stacking interactions [*Cg*2···*Cg*3 = 3.6380 (19) Å, symmetry code: -x+1, -y, -z+1 where *Cg*2 and *Cg*3 are the centroids of benzene (C14B-C19B) and 2*H*-pyran (C1B/O1B/C2B/C7B/C8B/C9B) rings].

### Experimental

3-Hydroxybenzaldehyde thiosemicarbazone (Lv *et al.*, 2010) and 3-[ω-bromoacetyl coumarin] (Siddiqui *et al.*, 2009) were synthesized as reported in the literatures. A solution of 3-[ω-bromoacetyl coumarin] (2.5 mmol) and 3-hydroxybenzaldehyde thiosemicarbazone (2.5 mmol) in chloroform-ethanol (2:1) was refluxed for 1 h at 353 K. A clear solution was formed followed by the deposition of thick yellow precipitates. The reaction mixture was cooled and basified with ammonia. The title compound, (I) was purified and recrystallized as shiny yellow plates of (I) from ethanol-ethyl acetate (1:2); the water of crystallisation was presumably incorporated from the atmosphere.

### Refinement

The H atoms bound to O and N atoms were located from the difference Fourier map and constrained to ride with the parent atom with *U*_iso_ = 1.5 *U*_eq_(O) or *U*_iso_ = 1.2 *U*_eq_(N). All other H atoms were placed in their calculated positions, with C—H = 0.93 Å, and refined using a riding model, with *U*_iso_ = 1.2 *U*_eq_(C).

### Crystal data

**Table:**

C_19_H_13_N_3_O_3_S·0.5H_2_O	*F*(000) = 1544
*M**_r_* = 372.39	*D*_x_ = 1.495 Mg m^−^^3^
Monoclinic, *P*2_1_/*c*	Mo *K*α radiation, λ = 0.71073 Å
Hall symbol: -P 2ybc	Cell parameters from 3134 reflections
*a* = 8.012 (3) Å	θ = 2.5–27.6°
*b* = 32.775 (11) Å	µ = 0.23 mm^−^^1^
*c* = 12.619 (4) Å	*T* = 100 K
β = 93.034 (7)°	Plate, yellow
*V* = 3309 (2) Å^3^	0.34 × 0.14 × 0.05 mm
*Z* = 8	

### Data collection

**Table:**

Bruker APEXII DUO CCD diffractometer	7564 independent reflections
Radiation source: fine-focus sealed tube	5266 reflections with *I* > 2σ(*I*)
graphite	*R*_int_ = 0.073
φ and ω scans	θ_max_ = 27.5°, θ_min_ = 2.5°
Absorption correction: multi-scan (*SADABS*; Bruker, 2009)	*h* = −10→10
*T*_min_ = 0.928, *T*_max_ = 0.990	*k* = −42→42
31194 measured reflections	*l* = −16→16

### Refinement

**Table:**

Refinement on *F*^2^	Primary atom site location: structure-invariant direct methods
Least-squares matrix: full	Secondary atom site location: difference Fourier map
*R*[*F*^2^ > 2σ(*F*^2^)] = 0.048	Hydrogen site location: inferred from neighbouring sites
*wR*(*F*^2^) = 0.128	H-atom parameters constrained
*S* = 1.06	*w* = 1/[σ^2^(*F*_o_^2^) + (0.0407*P*)^2^ + 2.6662*P*] where *P* = (*F*_o_^2^ + 2*F*_c_^2^)/3
7564 reflections	(Δ/σ)_max_ < 0.001
480 parameters	Δρ_max_ = 0.94 e Å^−^^3^
0 restraints	Δρ_min_ = −0.31 e Å^−^^3^

### Special details

**Table:**

Experimental. The crystal was placed in the cold stream of an Oxford Cryosystems Cobra open-flow nitrogen cryostat (Cosier & Glazer, 1986) operating at 100.0 (1)K.
Geometry. All esds (except the esd in the dihedral angle between two l.s. planes) are estimated using the full covariance matrix. The cell esds are taken into account individually in the estimation of esds in distances, angles and torsion angles; correlations between esds in cell parameters are only used when they are defined by crystal symmetry. An approximate (isotropic) treatment of cell esds is used for estimating esds involving l.s. planes.
Refinement. Refinement of F^2^ against ALL reflections. The weighted R-factor wR and goodness of fit S are based on F^2^, conventional R-factors R are based on F, with F set to zero for negative F^2^. The threshold expression of F^2^ > 2sigma(F^2^) is used only for calculating R-factors(gt) etc. and is not relevant to the choice of reflections for refinement. R-factors based on F^2^ are statistically about twice as large as those based on F, and R- factors based on ALL data will be even larger.

### Fractional atomic coordinates and isotropic or equivalent isotropic displacement parameters (Å^2^)

**Table:**

	*x*	*y*	*z*	*U*_iso_*/*U*_eq_	
S1A	−0.04957 (8)	0.042270 (18)	0.83927 (5)	0.01871 (15)	
O1A	−0.0143 (2)	−0.12215 (5)	0.63046 (14)	0.0206 (4)	
O2A	−0.1831 (2)	−0.07185 (5)	0.65803 (15)	0.0254 (4)	
O3A	−0.0075 (3)	0.22741 (6)	0.90542 (17)	0.0346 (5)	
H3OA	−0.0231	0.2112	0.8563	0.052*	
N1A	0.1639 (2)	−0.01536 (6)	0.87974 (16)	0.0154 (4)	
N2A	0.2018 (3)	0.04304 (6)	0.98605 (16)	0.0170 (4)	
H2NA	0.2795	0.0332	1.0306	0.020*	
N3A	0.1505 (3)	0.08281 (6)	0.99354 (17)	0.0175 (4)	
C1A	−0.0463 (3)	−0.08587 (7)	0.6792 (2)	0.0203 (5)	
C2A	0.1305 (3)	−0.14405 (7)	0.6527 (2)	0.0185 (5)	
C3A	0.1447 (3)	−0.18124 (8)	0.6037 (2)	0.0231 (6)	
H3A	0.0598	−0.1910	0.5573	0.028*	
C4A	0.2874 (3)	−0.20371 (8)	0.6249 (2)	0.0242 (6)	
H4A	0.2986	−0.2291	0.5933	0.029*	
C5A	0.4147 (3)	−0.18901 (8)	0.6929 (2)	0.0242 (6)	
H5A	0.5118	−0.2042	0.7052	0.029*	
C6A	0.3980 (3)	−0.15206 (7)	0.7422 (2)	0.0203 (5)	
H6A	0.4832	−0.1425	0.7886	0.024*	
C7A	0.2529 (3)	−0.12857 (7)	0.7231 (2)	0.0175 (5)	
C8A	0.2251 (3)	−0.08987 (7)	0.7713 (2)	0.0181 (5)	
H8A	0.3072	−0.0791	0.8180	0.022*	
C9A	0.0829 (3)	−0.06862 (7)	0.75075 (19)	0.0159 (5)	
C10A	0.0537 (3)	−0.02896 (7)	0.79832 (19)	0.0153 (5)	
C11A	−0.0693 (3)	−0.00203 (7)	0.7687 (2)	0.0182 (5)	
H11A	−0.1532	−0.0071	0.7166	0.022*	
C12A	0.1223 (3)	0.02122 (7)	0.90784 (19)	0.0167 (5)	
C13A	0.2049 (3)	0.10343 (7)	1.0744 (2)	0.0183 (5)	
H13A	0.2727	0.0914	1.1278	0.022*	
C14A	0.1576 (3)	0.14634 (7)	1.0806 (2)	0.0182 (5)	
C15A	0.1798 (3)	0.16766 (8)	1.1748 (2)	0.0220 (6)	
H15A	0.2258	0.1548	1.2352	0.026*	
C16A	0.1327 (3)	0.20862 (8)	1.1787 (2)	0.0286 (6)	
H16A	0.1441	0.2228	1.2424	0.034*	
C17A	0.0696 (3)	0.22810 (8)	1.0886 (2)	0.0277 (6)	
H17A	0.0395	0.2555	1.0914	0.033*	
C18A	0.0509 (3)	0.20689 (8)	0.9942 (2)	0.0256 (6)	
C19A	0.0919 (3)	0.16639 (7)	0.9902 (2)	0.0207 (5)	
H19A	0.0759	0.1521	0.9267	0.025*	
S1B	0.78931 (7)	0.008872 (18)	0.47478 (5)	0.01707 (14)	
O1B	0.7053 (2)	−0.14603 (5)	0.21875 (14)	0.0202 (4)	
O2B	0.8716 (2)	−0.11627 (5)	0.33656 (15)	0.0234 (4)	
O3B	0.9374 (2)	0.18069 (5)	0.72037 (16)	0.0260 (4)	
H3OB	0.9854	0.1590	0.7115	0.039*	
N1B	0.5592 (2)	−0.00941 (6)	0.33149 (16)	0.0159 (4)	
N2B	0.5221 (3)	0.05370 (6)	0.41319 (16)	0.0174 (4)	
H2NB	0.4267	0.0584	0.3851	0.021*	
N3B	0.5912 (3)	0.08171 (6)	0.48285 (16)	0.0162 (4)	
C1B	0.7465 (3)	−0.11277 (7)	0.2789 (2)	0.0182 (5)	
C2B	0.5703 (3)	−0.14700 (7)	0.14717 (19)	0.0175 (5)	
C3B	0.5399 (3)	−0.18320 (8)	0.0931 (2)	0.0224 (6)	
H3B	0.6058	−0.2061	0.1073	0.027*	
C4B	0.4100 (3)	−0.18436 (8)	0.0180 (2)	0.0241 (6)	
H4B	0.3878	−0.2084	−0.0195	0.029*	
C5B	0.3109 (3)	−0.15024 (8)	−0.0028 (2)	0.0219 (6)	
H5B	0.2235	−0.1515	−0.0543	0.026*	
C6B	0.3415 (3)	−0.11466 (8)	0.0523 (2)	0.0214 (5)	
H6B	0.2751	−0.0919	0.0379	0.026*	
C7B	0.4727 (3)	−0.11258 (7)	0.13051 (19)	0.0174 (5)	
C8B	0.5100 (3)	−0.07779 (7)	0.19488 (19)	0.0177 (5)	
H8B	0.4432	−0.0547	0.1860	0.021*	
C9B	0.6387 (3)	−0.07698 (7)	0.26843 (19)	0.0153 (5)	
C10B	0.6726 (3)	−0.04195 (7)	0.33772 (19)	0.0156 (5)	
C11B	0.8019 (3)	−0.03722 (7)	0.4095 (2)	0.0176 (5)	
H11B	0.8866	−0.0563	0.4222	0.021*	
C12B	0.6075 (3)	0.01856 (7)	0.39946 (19)	0.0152 (5)	
C13B	0.4999 (3)	0.11281 (7)	0.50071 (19)	0.0171 (5)	
H13B	0.3917	0.1141	0.4703	0.021*	
C14B	0.5629 (3)	0.14618 (7)	0.56794 (19)	0.0165 (5)	
C15B	0.4627 (3)	0.18039 (7)	0.5765 (2)	0.0201 (5)	
H15B	0.3557	0.1808	0.5441	0.024*	
C16B	0.5224 (3)	0.21406 (7)	0.6334 (2)	0.0215 (5)	
H16B	0.4549	0.2369	0.6400	0.026*	
C17B	0.6821 (3)	0.21366 (7)	0.6802 (2)	0.0212 (5)	
H17B	0.7234	0.2365	0.7166	0.025*	
C18B	0.7808 (3)	0.17906 (7)	0.6729 (2)	0.0187 (5)	
C19B	0.7216 (3)	0.14544 (7)	0.61789 (19)	0.0174 (5)	
H19B	0.7877	0.1222	0.6141	0.021*	
O1W	0.5255 (2)	0.97623 (5)	0.87645 (14)	0.0220 (4)	
H1W1	0.5231	0.9845	0.8111	0.033*	
H2W1	0.4267	0.9713	0.9003	0.033*	

### Atomic displacement parameters (Å^2^)

**Table:**

	*U*^11^	*U*^22^	*U*^33^	*U*^12^	*U*^13^	*U*^23^
S1A	0.0181 (3)	0.0184 (3)	0.0194 (3)	0.0046 (2)	−0.0013 (2)	−0.0010 (2)
O1A	0.0233 (10)	0.0184 (9)	0.0196 (9)	−0.0026 (7)	−0.0022 (8)	−0.0040 (7)
O2A	0.0188 (9)	0.0264 (10)	0.0299 (11)	0.0018 (8)	−0.0097 (8)	−0.0033 (8)
O3A	0.0465 (13)	0.0223 (10)	0.0337 (12)	0.0053 (9)	−0.0083 (10)	0.0015 (9)
N1A	0.0152 (10)	0.0162 (10)	0.0148 (10)	−0.0007 (8)	−0.0002 (8)	0.0012 (8)
N2A	0.0190 (11)	0.0142 (10)	0.0173 (10)	0.0047 (8)	−0.0035 (8)	−0.0021 (8)
N3A	0.0172 (10)	0.0142 (10)	0.0212 (11)	0.0028 (8)	0.0017 (9)	−0.0015 (8)
C1A	0.0241 (14)	0.0181 (12)	0.0184 (12)	−0.0017 (10)	−0.0003 (11)	0.0006 (10)
C2A	0.0226 (13)	0.0169 (12)	0.0164 (12)	0.0000 (10)	0.0046 (10)	0.0026 (10)
C3A	0.0300 (15)	0.0210 (13)	0.0184 (13)	−0.0081 (11)	0.0033 (11)	−0.0020 (11)
C4A	0.0351 (16)	0.0155 (12)	0.0228 (14)	−0.0028 (11)	0.0088 (12)	−0.0023 (11)
C5A	0.0290 (15)	0.0182 (12)	0.0259 (14)	0.0053 (11)	0.0062 (12)	0.0032 (11)
C6A	0.0224 (13)	0.0185 (12)	0.0202 (13)	0.0024 (10)	0.0016 (11)	0.0008 (10)
C7A	0.0221 (13)	0.0148 (11)	0.0158 (12)	−0.0014 (10)	0.0019 (10)	0.0019 (10)
C8A	0.0181 (12)	0.0184 (12)	0.0176 (12)	−0.0014 (10)	−0.0019 (10)	−0.0005 (10)
C9A	0.0166 (12)	0.0169 (11)	0.0143 (11)	−0.0018 (10)	−0.0001 (10)	0.0032 (9)
C10A	0.0134 (12)	0.0181 (11)	0.0146 (11)	−0.0010 (9)	0.0022 (9)	0.0010 (10)
C11A	0.0171 (12)	0.0192 (12)	0.0180 (12)	−0.0002 (10)	−0.0011 (10)	−0.0010 (10)
C12A	0.0143 (12)	0.0184 (12)	0.0174 (12)	0.0005 (9)	0.0016 (10)	0.0021 (10)
C13A	0.0184 (13)	0.0177 (12)	0.0188 (13)	0.0010 (10)	0.0022 (10)	0.0006 (10)
C14A	0.0141 (12)	0.0158 (12)	0.0249 (13)	−0.0017 (9)	0.0035 (10)	0.0005 (10)
C15A	0.0214 (13)	0.0231 (13)	0.0217 (13)	−0.0028 (11)	0.0037 (11)	−0.0009 (11)
C16A	0.0296 (15)	0.0244 (14)	0.0328 (16)	−0.0067 (12)	0.0102 (13)	−0.0140 (12)
C17A	0.0278 (15)	0.0157 (12)	0.0400 (17)	0.0044 (11)	0.0050 (13)	−0.0044 (12)
C18A	0.0220 (14)	0.0204 (13)	0.0341 (16)	0.0018 (11)	−0.0020 (12)	0.0007 (12)
C19A	0.0155 (12)	0.0197 (12)	0.0270 (14)	−0.0026 (10)	0.0004 (11)	0.0017 (11)
S1B	0.0155 (3)	0.0181 (3)	0.0173 (3)	0.0001 (2)	−0.0027 (2)	−0.0011 (2)
O1B	0.0229 (9)	0.0144 (8)	0.0230 (9)	0.0012 (7)	−0.0023 (8)	−0.0020 (7)
O2B	0.0223 (10)	0.0209 (9)	0.0263 (10)	0.0016 (8)	−0.0055 (8)	0.0001 (8)
O3B	0.0257 (10)	0.0202 (9)	0.0310 (11)	0.0042 (8)	−0.0091 (9)	−0.0059 (8)
N1B	0.0148 (10)	0.0161 (10)	0.0168 (10)	0.0014 (8)	0.0012 (8)	−0.0015 (8)
N2B	0.0154 (10)	0.0160 (10)	0.0202 (11)	0.0025 (8)	−0.0029 (9)	−0.0037 (8)
N3B	0.0194 (11)	0.0148 (10)	0.0143 (10)	−0.0030 (8)	0.0005 (8)	−0.0020 (8)
C1B	0.0208 (13)	0.0160 (12)	0.0180 (12)	−0.0020 (10)	0.0025 (10)	0.0013 (10)
C2B	0.0159 (12)	0.0210 (12)	0.0158 (12)	−0.0048 (10)	0.0032 (10)	−0.0002 (10)
C3B	0.0275 (14)	0.0176 (12)	0.0227 (13)	−0.0019 (11)	0.0050 (11)	−0.0016 (11)
C4B	0.0288 (15)	0.0220 (13)	0.0221 (13)	−0.0077 (11)	0.0071 (12)	−0.0047 (11)
C5B	0.0184 (13)	0.0276 (13)	0.0200 (13)	−0.0049 (11)	0.0031 (11)	−0.0068 (11)
C6B	0.0181 (13)	0.0264 (13)	0.0199 (13)	−0.0010 (11)	0.0015 (10)	−0.0018 (11)
C7B	0.0166 (12)	0.0183 (12)	0.0178 (12)	−0.0024 (10)	0.0040 (10)	0.0000 (10)
C8B	0.0182 (12)	0.0162 (11)	0.0189 (12)	0.0013 (10)	0.0012 (10)	0.0000 (10)
C9B	0.0145 (12)	0.0145 (11)	0.0174 (12)	−0.0010 (9)	0.0035 (10)	0.0005 (9)
C10B	0.0169 (12)	0.0147 (11)	0.0151 (12)	−0.0006 (9)	0.0016 (10)	0.0000 (9)
C11B	0.0159 (12)	0.0161 (11)	0.0206 (12)	0.0010 (10)	0.0005 (10)	0.0013 (10)
C12B	0.0136 (11)	0.0162 (11)	0.0158 (12)	−0.0013 (9)	0.0010 (9)	0.0017 (9)
C13B	0.0149 (12)	0.0180 (12)	0.0183 (12)	−0.0016 (10)	0.0000 (10)	0.0025 (10)
C14B	0.0209 (12)	0.0141 (11)	0.0148 (12)	−0.0024 (10)	0.0043 (10)	0.0028 (9)
C15B	0.0193 (13)	0.0187 (12)	0.0226 (13)	−0.0006 (10)	0.0029 (11)	0.0004 (10)
C16B	0.0239 (13)	0.0153 (12)	0.0258 (14)	0.0030 (10)	0.0059 (11)	−0.0011 (10)
C17B	0.0265 (14)	0.0143 (12)	0.0229 (13)	−0.0011 (10)	0.0028 (11)	−0.0014 (10)
C18B	0.0208 (13)	0.0179 (12)	0.0170 (12)	−0.0006 (10)	−0.0030 (10)	0.0009 (10)
C19B	0.0209 (13)	0.0137 (11)	0.0179 (12)	0.0015 (10)	0.0029 (10)	0.0018 (10)
O1W	0.0188 (9)	0.0284 (10)	0.0186 (9)	0.0012 (8)	−0.0019 (7)	0.0033 (8)

### Geometric parameters (Å, °)

**Table:**

S1A—C11A	1.706 (2)	S1B—C12B	1.726 (2)
S1A—C12A	1.730 (2)	O1B—C1B	1.359 (3)
O1A—C1A	1.369 (3)	O1B—C2B	1.372 (3)
O1A—C2A	1.380 (3)	O2B—C1B	1.212 (3)
O2A—C1A	1.206 (3)	O3B—C18B	1.362 (3)
O3A—C18A	1.367 (3)	O3B—H3OB	0.8200
O3A—H3OA	0.8200	N1B—C12B	1.300 (3)
N1A—C12A	1.299 (3)	N1B—C10B	1.400 (3)
N1A—C10A	1.392 (3)	N2B—C12B	1.355 (3)
N2A—C12A	1.351 (3)	N2B—N3B	1.367 (3)
N2A—N3A	1.372 (3)	N2B—H2NB	0.8398
N2A—H2NA	0.8779	N3B—C13B	1.281 (3)
N3A—C13A	1.281 (3)	C1B—C9B	1.459 (3)
C1A—C9A	1.452 (3)	C2B—C7B	1.382 (3)
C2A—C3A	1.374 (3)	C2B—C3B	1.384 (3)
C2A—C7A	1.385 (4)	C3B—C4B	1.371 (4)
C3A—C4A	1.375 (4)	C3B—H3B	0.9300
C3A—H3A	0.9300	C4B—C5B	1.389 (4)
C4A—C5A	1.384 (4)	C4B—H4B	0.9300
C4A—H4A	0.9300	C5B—C6B	1.374 (3)
C5A—C6A	1.371 (3)	C5B—H5B	0.9300
C5A—H5A	0.9300	C6B—C7B	1.404 (4)
C6A—C7A	1.404 (3)	C6B—H6B	0.9300
C6A—H6A	0.9300	C7B—C8B	1.423 (3)
C7A—C8A	1.429 (3)	C8B—C9B	1.350 (3)
C8A—C9A	1.348 (3)	C8B—H8B	0.9300
C8A—H8A	0.9300	C9B—C10B	1.460 (3)
C9A—C10A	1.456 (3)	C10B—C11B	1.348 (3)
C10A—C11A	1.361 (3)	C11B—H11B	0.9300
C11A—H11A	0.9300	C13B—C14B	1.458 (3)
C13A—C14A	1.460 (3)	C13B—H13B	0.9300
C13A—H13A	0.9300	C14B—C15B	1.386 (3)
C14A—C15A	1.383 (4)	C14B—C19B	1.389 (3)
C14A—C19A	1.395 (4)	C15B—C16B	1.388 (3)
C15A—C16A	1.396 (4)	C15B—H15B	0.9300
C15A—H15A	0.9300	C16B—C17B	1.380 (4)
C16A—C17A	1.376 (4)	C16B—H16B	0.9300
C16A—H16A	0.9300	C17B—C18B	1.389 (3)
C17A—C18A	1.381 (4)	C17B—H17B	0.9300
C17A—H17A	0.9300	C18B—C19B	1.373 (3)
C18A—C19A	1.369 (3)	C19B—H19B	0.9300
C19A—H19A	0.9300	O1W—H1W1	0.8672
S1B—C11B	1.726 (2)	O1W—H2W1	0.8763
			
C11A—S1A—C12A	88.33 (12)	C1B—O1B—C2B	123.10 (19)
C1A—O1A—C2A	122.4 (2)	C18B—O3B—H3OB	109.5
C18A—O3A—H3OA	109.5	C12B—N1B—C10B	109.3 (2)
C12A—N1A—C10A	109.6 (2)	C12B—N2B—N3B	117.6 (2)
C12A—N2A—N3A	114.9 (2)	C12B—N2B—H2NB	123.8
C12A—N2A—H2NA	124.4	N3B—N2B—H2NB	118.5
N3A—N2A—H2NA	120.7	C13B—N3B—N2B	115.6 (2)
C13A—N3A—N2A	117.7 (2)	O2B—C1B—O1B	115.4 (2)
O2A—C1A—O1A	114.9 (2)	O2B—C1B—C9B	126.6 (2)
O2A—C1A—C9A	126.8 (2)	O1B—C1B—C9B	118.0 (2)
O1A—C1A—C9A	118.3 (2)	O1B—C2B—C7B	120.1 (2)
C3A—C2A—O1A	117.3 (2)	O1B—C2B—C3B	117.2 (2)
C3A—C2A—C7A	122.7 (2)	C7B—C2B—C3B	122.8 (2)
O1A—C2A—C7A	119.9 (2)	C4B—C3B—C2B	118.2 (2)
C2A—C3A—C4A	118.4 (2)	C4B—C3B—H3B	120.9
C2A—C3A—H3A	120.8	C2B—C3B—H3B	120.9
C4A—C3A—H3A	120.8	C3B—C4B—C5B	121.0 (2)
C3A—C4A—C5A	120.8 (2)	C3B—C4B—H4B	119.5
C3A—C4A—H4A	119.6	C5B—C4B—H4B	119.5
C5A—C4A—H4A	119.6	C6B—C5B—C4B	120.2 (3)
C6A—C5A—C4A	120.1 (2)	C6B—C5B—H5B	119.9
C6A—C5A—H5A	119.9	C4B—C5B—H5B	119.9
C4A—C5A—H5A	119.9	C5B—C6B—C7B	120.3 (2)
C5A—C6A—C7A	120.4 (2)	C5B—C6B—H6B	119.9
C5A—C6A—H6A	119.8	C7B—C6B—H6B	119.9
C7A—C6A—H6A	119.8	C2B—C7B—C6B	117.6 (2)
C2A—C7A—C6A	117.5 (2)	C2B—C7B—C8B	117.8 (2)
C2A—C7A—C8A	118.5 (2)	C6B—C7B—C8B	124.5 (2)
C6A—C7A—C8A	124.0 (2)	C9B—C8B—C7B	122.5 (2)
C9A—C8A—C7A	121.7 (2)	C9B—C8B—H8B	118.7
C9A—C8A—H8A	119.1	C7B—C8B—H8B	118.7
C7A—C8A—H8A	119.1	C8B—C9B—C1B	118.2 (2)
C8A—C9A—C1A	119.0 (2)	C8B—C9B—C10B	122.6 (2)
C8A—C9A—C10A	122.3 (2)	C1B—C9B—C10B	119.2 (2)
C1A—C9A—C10A	118.7 (2)	C11B—C10B—N1B	115.0 (2)
C11A—C10A—N1A	114.6 (2)	C11B—C10B—C9B	127.2 (2)
C11A—C10A—C9A	126.6 (2)	N1B—C10B—C9B	117.7 (2)
N1A—C10A—C9A	118.7 (2)	C10B—C11B—S1B	111.06 (18)
C10A—C11A—S1A	111.28 (19)	C10B—C11B—H11B	124.5
C10A—C11A—H11A	124.4	S1B—C11B—H11B	124.5
S1A—C11A—H11A	124.4	N1B—C12B—N2B	123.3 (2)
N1A—C12A—N2A	124.7 (2)	N1B—C12B—S1B	116.50 (18)
N1A—C12A—S1A	116.15 (18)	N2B—C12B—S1B	120.19 (18)
N2A—C12A—S1A	119.10 (18)	N3B—C13B—C14B	121.1 (2)
N3A—C13A—C14A	118.2 (2)	N3B—C13B—H13B	119.5
N3A—C13A—H13A	120.9	C14B—C13B—H13B	119.5
C14A—C13A—H13A	120.9	C15B—C14B—C19B	119.9 (2)
C15A—C14A—C19A	119.4 (2)	C15B—C14B—C13B	117.9 (2)
C15A—C14A—C13A	120.7 (2)	C19B—C14B—C13B	122.1 (2)
C19A—C14A—C13A	119.9 (2)	C14B—C15B—C16B	119.9 (2)
C14A—C15A—C16A	119.6 (3)	C14B—C15B—H15B	120.1
C14A—C15A—H15A	120.2	C16B—C15B—H15B	120.1
C16A—C15A—H15A	120.2	C17B—C16B—C15B	120.0 (2)
C17A—C16A—C15A	120.4 (3)	C17B—C16B—H16B	120.0
C17A—C16A—H16A	119.8	C15B—C16B—H16B	120.0
C15A—C16A—H16A	119.8	C16B—C17B—C18B	119.8 (2)
C16A—C17A—C18A	119.8 (2)	C16B—C17B—H17B	120.1
C16A—C17A—H17A	120.1	C18B—C17B—H17B	120.1
C18A—C17A—H17A	120.1	O3B—C18B—C19B	122.6 (2)
O3A—C18A—C19A	121.3 (3)	O3B—C18B—C17B	117.0 (2)
O3A—C18A—C17A	118.4 (2)	C19B—C18B—C17B	120.4 (2)
C19A—C18A—C17A	120.3 (3)	C18B—C19B—C14B	119.9 (2)
C18A—C19A—C14A	120.5 (3)	C18B—C19B—H19B	120.0
C18A—C19A—H19A	119.7	C14B—C19B—H19B	120.0
C14A—C19A—H19A	119.7	H1W1—O1W—H2W1	114.1
C11B—S1B—C12B	88.11 (12)		
			
C12A—N2A—N3A—C13A	170.9 (2)	C12B—N2B—N3B—C13B	−174.0 (2)
C2A—O1A—C1A—O2A	−174.4 (2)	C2B—O1B—C1B—O2B	−178.3 (2)
C2A—O1A—C1A—C9A	4.7 (3)	C2B—O1B—C1B—C9B	1.8 (3)
C1A—O1A—C2A—C3A	176.2 (2)	C1B—O1B—C2B—C7B	2.5 (3)
C1A—O1A—C2A—C7A	−2.9 (3)	C1B—O1B—C2B—C3B	−178.3 (2)
O1A—C2A—C3A—C4A	−179.7 (2)	O1B—C2B—C3B—C4B	−177.3 (2)
C7A—C2A—C3A—C4A	−0.7 (4)	C7B—C2B—C3B—C4B	1.8 (4)
C2A—C3A—C4A—C5A	−0.9 (4)	C2B—C3B—C4B—C5B	−0.4 (4)
C3A—C4A—C5A—C6A	1.6 (4)	C3B—C4B—C5B—C6B	−0.3 (4)
C4A—C5A—C6A—C7A	−0.9 (4)	C4B—C5B—C6B—C7B	−0.3 (4)
C3A—C2A—C7A—C6A	1.3 (4)	O1B—C2B—C7B—C6B	176.7 (2)
O1A—C2A—C7A—C6A	−179.7 (2)	C3B—C2B—C7B—C6B	−2.4 (4)
C3A—C2A—C7A—C8A	−179.0 (2)	O1B—C2B—C7B—C8B	−4.4 (3)
O1A—C2A—C7A—C8A	0.1 (4)	C3B—C2B—C7B—C8B	176.6 (2)
C5A—C6A—C7A—C2A	−0.5 (4)	C5B—C6B—C7B—C2B	1.6 (4)
C5A—C6A—C7A—C8A	179.8 (2)	C5B—C6B—C7B—C8B	−177.3 (2)
C2A—C7A—C8A—C9A	0.8 (4)	C2B—C7B—C8B—C9B	1.9 (4)
C6A—C7A—C8A—C9A	−179.5 (2)	C6B—C7B—C8B—C9B	−179.3 (2)
C7A—C8A—C9A—C1A	1.1 (4)	C7B—C8B—C9B—C1B	2.3 (4)
C7A—C8A—C9A—C10A	−179.3 (2)	C7B—C8B—C9B—C10B	−177.2 (2)
O2A—C1A—C9A—C8A	175.3 (3)	O2B—C1B—C9B—C8B	175.9 (2)
O1A—C1A—C9A—C8A	−3.7 (3)	O1B—C1B—C9B—C8B	−4.2 (3)
O2A—C1A—C9A—C10A	−4.4 (4)	O2B—C1B—C9B—C10B	−4.5 (4)
O1A—C1A—C9A—C10A	176.6 (2)	O1B—C1B—C9B—C10B	175.4 (2)
C12A—N1A—C10A—C11A	−0.7 (3)	C12B—N1B—C10B—C11B	−0.3 (3)
C12A—N1A—C10A—C9A	176.8 (2)	C12B—N1B—C10B—C9B	179.0 (2)
C8A—C9A—C10A—C11A	167.1 (2)	C8B—C9B—C10B—C11B	−176.3 (2)
C1A—C9A—C10A—C11A	−13.3 (4)	C1B—C9B—C10B—C11B	4.1 (4)
C8A—C9A—C10A—N1A	−10.1 (4)	C8B—C9B—C10B—N1B	4.5 (3)
C1A—C9A—C10A—N1A	169.6 (2)	C1B—C9B—C10B—N1B	−175.1 (2)
N1A—C10A—C11A—S1A	1.5 (3)	N1B—C10B—C11B—S1B	0.1 (3)
C9A—C10A—C11A—S1A	−175.7 (2)	C9B—C10B—C11B—S1B	−179.1 (2)
C12A—S1A—C11A—C10A	−1.42 (19)	C12B—S1B—C11B—C10B	0.09 (19)
C10A—N1A—C12A—N2A	178.9 (2)	C10B—N1B—C12B—N2B	−179.2 (2)
C10A—N1A—C12A—S1A	−0.4 (3)	C10B—N1B—C12B—S1B	0.4 (3)
N3A—N2A—C12A—N1A	172.1 (2)	N3B—N2B—C12B—N1B	−176.3 (2)
N3A—N2A—C12A—S1A	−8.7 (3)	N3B—N2B—C12B—S1B	4.2 (3)
C11A—S1A—C12A—N1A	1.1 (2)	C11B—S1B—C12B—N1B	−0.3 (2)
C11A—S1A—C12A—N2A	−178.2 (2)	C11B—S1B—C12B—N2B	179.3 (2)
N2A—N3A—C13A—C14A	177.0 (2)	N2B—N3B—C13B—C14B	−176.0 (2)
N3A—C13A—C14A—C15A	165.4 (2)	N3B—C13B—C14B—C15B	174.6 (2)
N3A—C13A—C14A—C19A	−15.7 (3)	N3B—C13B—C14B—C19B	−1.8 (4)
C19A—C14A—C15A—C16A	1.4 (4)	C19B—C14B—C15B—C16B	1.1 (4)
C13A—C14A—C15A—C16A	−179.7 (2)	C13B—C14B—C15B—C16B	−175.4 (2)
C14A—C15A—C16A—C17A	−2.1 (4)	C14B—C15B—C16B—C17B	0.9 (4)
C15A—C16A—C17A—C18A	0.7 (4)	C15B—C16B—C17B—C18B	−1.9 (4)
C16A—C17A—C18A—O3A	−178.1 (3)	C16B—C17B—C18B—O3B	179.7 (2)
C16A—C17A—C18A—C19A	1.2 (4)	C16B—C17B—C18B—C19B	1.1 (4)
O3A—C18A—C19A—C14A	177.4 (2)	O3B—C18B—C19B—C14B	−177.7 (2)
C17A—C18A—C19A—C14A	−1.9 (4)	C17B—C18B—C19B—C14B	0.9 (4)
C15A—C14A—C19A—C18A	0.5 (4)	C15B—C14B—C19B—C18B	−2.0 (4)
C13A—C14A—C19A—C18A	−178.4 (2)	C13B—C14B—C19B—C18B	174.4 (2)

### Hydrogen-bond geometry (Å, °)

**Table:**

Cg1 is the centroid of C14A–C19A benzene ring.

**Table:**

*D*—H···*A*	*D*—H	H···*A*	*D*···*A*	*D*—H···*A*
O3A—H3OA···O3B^i^	0.82	2.00	2.808 (3)	170
N2A—H2NA···O1W^ii^	0.88	1.93	2.790 (3)	167
O3B—H3OB···O2B^iii^	0.82	1.93	2.726 (3)	165
N2B—H2NB···O2A^iv^	0.84	2.05	2.878 (3)	170
O1W—H1W1···N1B^v^	0.87	2.05	2.888 (3)	161
O1W—H2W1···N1A^vi^	0.88	2.15	2.913 (3)	145
C8A—H8A···O1W^vii^	0.93	2.60	3.451 (3)	153
C5B—H5B···Cg1^iv^	0.93	2.95	3.708 (3)	139

Symmetry codes: (i) *x*−1, *y*, *z*; (ii) −*x*+1, −*y*+1, −*z*+2; (iii) −*x*+2, −*y*, −*z*+1; (iv) −*x*, −*y*, −*z*+1; (v) −*x*+1, −*y*+1, −*z*+1; (vi) *x*, *y*+1, *z*; (vii) *x*, *y*−1, *z*.
